# Strategy use and its evolvement in word list learning: a replication study

**DOI:** 10.1098/rsos.230651

**Published:** 2024-02-14

**Authors:** Matti Laine, Daniel Fellman, Tilda Eräste, Liisa Ritakallio, Juha Salmi

**Affiliations:** ^1^ Department of Psychology, Åbo Akademi University, Turku, Finland; ^2^ Department of Neuroscience and Biomedical Engineering, Aalto University, Espoo, Finland

**Keywords:** episodic memory, mnemonics, memory strategy, list learning, skill learning

## Abstract

Spontaneous strategy employment is important for memory performance, but systematic research on strategy use and within-task evolvement is limited. This online study aimed to replicate three main findings by Waris and colleagues in *Quarterly Journal of Experimental Psychology* (2021): in word-list learning, spontaneous strategy use (1) predicts better task performance, (2) stabilizes along the task, and (3) increases during the first two task blocks. We administered a shortened version of their original real-word list-learning task to 209 neurotypical adults. Their first finding was partly replicated: manipulation strategies (grouping, visualization, association, narrative, other strategy) but not maintenance strategies (rehearsal/repetition, selective focus) were associated with superior word recall. The second finding on the decrease in strategy changers over task blocks was replicated. The third finding turned out to be misguided: neither our nor the original study showed task-initial increase in strategy use in the real-word learning condition. Our results confirm the important role of spontaneous strategies in understanding memory performance and the existence of task-initial dynamics in strategy employment. They support the general conclusions by Waris and colleagues: task demands can trigger strategy use even in a familiar task like learning a list of common words, and evolution of strategy use during a memory task reflects cognitive skill learning.

## 1. Introduction

Strategy use is an important aspect of memory and learning that contributes to individual differences in performance [1,2]. Earlier studies have indicated that spontaneous strategy employment is associated with higher performance in different memory tasks [3–12]. However, the within-task dynamics of memory strategy use remain largely unexplored. Besides retrospective (i.e. after the memory task has been completed) strategy reports, also concurrent block-by-block or trial-by-trial strategy reports have been employed [13–15], but in most cases such data have not been used to examine possible changes in strategy use within a memory task. A few exceptions exist: Delaney and colleagues [16,17] reported spontaneous switching from more shallow self-reported strategies such as rote rehearsal to deeper strategies (e.g. making a story with to-be-remembered words) with increased practice with word lists. Their studies concerned specific experimental memory paradigms tapping directed forgetting and spaced encoding. Moreover, Unsworth *et al.* [18] found that in their word list recall task, high-performing participants exhibited more flexible strategy use where a less effective strategy employed on the first word list was replaced by a more effective one on the second list.

Establishing spontaneous evolvement of strategy use during a memory task is of interest as it links task performance to the general framework of cognitive skill learning. The skill learning view presupposes that performance on a new complex cognitive task is an adaptive process that can be divided into three major stages [19]. At the task-initial Formation stage, the metacognitive system establishes strategies and behavioural routines for successful task performance. At the Controlled Execution stage, these processes are effortfully put into use. This is followed by the final stage, Automatic Execution, during which performance becomes increasingly more automatic and modular. This means that the initial metacognitive and executive load becomes minimized and these resources become available to other tasks. In two recent studies, Waris *et al.* [11,12], using open strategy reports that should avoid the risk for reactivity effects in commonly used multiple-choice strategy measures [13], found within-task evolvement of strategy use in common episodic (word list learning) and working memory (n-back) tasks that is in line with the general cognitive skill learning framework. Such evolvement is of theoretical and practical interest as it reveals task-internal dynamics, taking place on the time-scale of minutes, which are not observable with the commonly employed summative memory test scores. As this phenomenon is only scarcely studied, we wanted to replicate and extend the results of Waris *et al*. [11] concerning the spontaneous strategy use in word list learning, an episodic memory task.

Waris *et al*. [11] employed two word list learning tasks, one with real words and the other with pseudowords, to examine the temporal pattern of strategy use and its role in recall performance in these tasks. Strategy use was probed by self-reports after each task block. In the present study, we used only the real word version that due to its relative familiarity to adult participants was critical to the hypotheses Waris *et al*. [11] tested. The central findings by Waris *et al*. [11] concerning the real word learning task—the findings we aimed to replicate—were as follows. (1) Strategy use was positively related to episodic memory performance. Based on earlier research [8], Waris *et al*. [11] re-coded the original strategy types into three major categories, namely No strategy, Maintenance (including the primary strategies of rehearsal and repetition)/Other strategy, and Manipulation (the memoranda were mentally manipulated for example through grouping, association or visualization). When using this categorization, they found that Manipulation strategy users performed better than Maintenance/Other strategy users across the task blocks. When compared to No strategy, Manipulation strategy users exhibited better performance and a steeper learning curve. The same pattern was observed when comparing No strategy to Maintenance/Other strategy. Another strategy variable, the level of strategy detail (how many strategy-related details were given in the open-ended strategy reports), was also associated with objective performance across the task. (2) The proportion of participants changing from one strategy to another between the blocks went down during the first block transitions. (3) Self-reported strategy use (dichotomized as yes/no) increased during the first two task blocks. The results (2) and (3) on the word learning task, indicating task-initial dynamics in strategy employment, were taken as support for the idea that memory task performance entails cognitive skill learning. Moreover, Waris *et al*. [11] concluded that familiarity with a task (learning a list of real words is a commonplace compared with pseudoword learning) does not necessarily lead to employment of readily available, stable strategies right from the start, as the cognitive routine framework proposed by Gathercole *et al*. [20] would suggest. Rather, Waris *et al*. [11] took this as support for the alternative task demand hypothesis, according to which memory demands posed by the task triggered task-initial strategy adjustment. As these effects were found only in a *post hoc* analysis focusing on the initial task blocks, it is important to try to replicate them.

In sum, this study attempted to replicate the following three central findings reported by Waris *et al*. [11]: strategy use in an episodic memory task (word list learning) predicts better objective task performance, strategic choices become more stable when the task advances, and strategy use increases during the first two task blocks. If these three findings are replicated, they would provide further support to the task demand hypothesis, according to which strategy generation is triggered by task difficulty rather than novelty (after all, learning a list of words can be considered as a rather familiar memory task for adults). At a more general level, successful replication would also provide support to the cognitive skill learning framework [19] by showing that even within the short time-span of a memory task, adaptive evolvement of strategy use takes place.

## 2. Methods

### 2.1. Participants and procedure

This article received results-blind in-principle acceptance (IPA) at *Royal Society Open Science*. Following IPA, the accepted Stage 1 version of the manuscript, not including results and discussion, was preregistered on the OSF (osf.io/j8vpy). This preregistration was performed after data analysis.

Ethics clearance for the study was obtained from the Ethics Board of the Departments of Psychology and Logopedics at the Åbo Akademi University, Turku, Finland. The data of the Word List Learning task that was used in the current study were collected as a part of a large-scale online experiment that has been published in a separate paper [21].

As in Waris *et al*. [11], recruitment took place via the crowdsourcing site Prolific (https://www.prolific.co/). Participants remained anonymous and received their monetary compensation via Prolific. The present study concerns only neurotypical participants, but the original study described in Jylkkä *et al*. [21] included also adults with diagnosed attention deficit hyperactivity disorder (ADHD) which explains the large initial screening samples. All participants were 18 to 50 years of age, lived in the UK and had English as their first language. Here we focused only on the neurotypical participants' performance and strategy use in the Word List Learning task that is described below.

The pre-registration of the larger project (https://osf.io/m7c9a) aimed at 250 neurotypical individuals who had completed the study. The motivation was that in this range correlations, an important analytical aspect for the larger project, stabilize [22]. While this decision did not take into account the strength of evidence obtained by Waris *et al*. [11], it should be noted that the present sample is twice the size of their sample of 101 individuals. To examine this further, we conducted additional analyses by altering the priors to assume large effects (*r* = 1) or small effects (*r* = 0.25). These were performed on our main analyses reported in the Results section, and the results consistently led to the same conclusions. This convergence across various prior assumptions reinforces the robustness of our findings and provides assurance that our conclusions would not be driven solely by a specific choice of priors.

**Figure 1.**
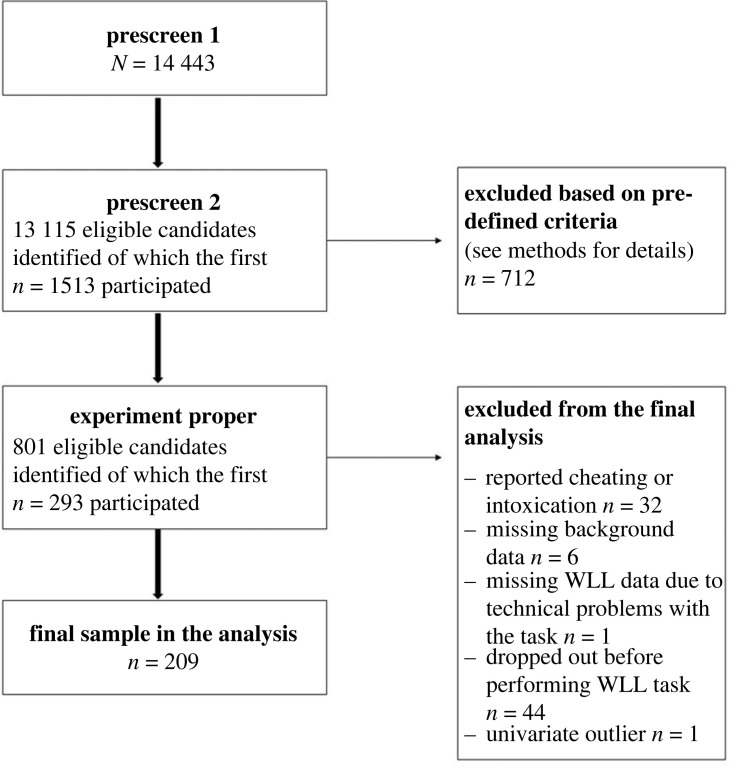
Flowchart of the data collection procedure. WLL = Word List Learning task.

The data were collected between August and December 2021. Data collection proceeded in three stages, with two short prescreening sessions and the actual study (figure 1 and Jylkkä *et al*. [21]). The aim of the first prescreening was to identify a large enough sample of adults with ADHD in the Prolific participant pool by posing a question on possible ADHD/ADD diagnosis and asking to fill out the Adult ADHD Self-Report Scale Part A [23]. At the next step, non-ADHD participants on a first-come-first-serve basis took part in the second prescreen (*N* = 1513) that probed basic demographics (age, gender, education, level of income); medical history (e.g. diagnosis of bipolar disorder, severe depression, psychosis, schizophrenia, or neurological illness); colour vision and eyesight; alcohol consumption with AUDIT questions 1–3; use of nicotine products; and use of other possible psychoactive substances. Furthermore, the participants filled out ASRS part B and DSM-5 Self-Rated Level 1 Cross-Cutting Symptom Measure—Adult [24], and some further questionnaires. The length of the second prescreening was about 10 min.

Next, 293 suitable participants took part in the study proper, again on a first-come-first-serve basis. The inclusion criteria were as follows: normal or corrected-to-normal vision; no colour blindness; no neurological illness that affects the participant's current life; no neurodevelopmental disorders; never diagnosed with bipolar disorder, severe depression, psychosis, or schizophrenia; and no self-reported problem with substance abuse. Additionally, they had to fulfil the following criteria in the DSM-5 Symptom Measure: no reported suicidality (score 0 on item 11) and sum scores less than three in the domains depression, mania, and anxiety. This means symptoms rated as ‘mild’, or symptom occurrence not more often than during ‘several days’ within the last 2-week period.

After considering all inclusion and exclusion criteria, missing data in our strategy and performance variables on the Word List Learning task (we performed listwise exclusions so that participants having one or more missing values were excluded), and univariate outlier screenings, the final sample size of the study group in the current analyses was 209. Thus, the present sample of neurotypical adults was twice the size of the group to which the present results were compared, i.e. the Repeated Strategy Queries group (*n* = 101) in Waris *et al*. [11]. That group in Waris *et al*. [11] was quite similar to ours in terms of sex (71% versus 75% females), average age (34.8 years versus 31.8 years), and distribution of educational attainment (66.1% versus 65.4% with bachelor's degree or higher). Table 1 depicts the background characteristics of the present final sample.

**Table 1.** Background characteristics of the sample *(n* = 209).

variable	distribution
gender (F/M/other)	157/52/0
age (*M*, s.d.)	31.82 (8.67)
education (%)	lower secondary 1.4
	higher secondary 17.2
	basic vocational 5.3
	vocational university 10.1
	bachelor's degree 45.5
	master's degree 18.2
	doctoral degree 2.4

### 2.2. Test sessions in the study proper

The actual study encompassed five separate online assessment sessions. It included three prospective memory tasks, questionnaires, a 5-day diary on everyday prospective memory lapses, and the Word List Learning task. One of the prospective memory tasks, the video game EPELI [21], was always in the first session, and the diary task was presented in each session. The presentation of all the other tasks, including Word List Learning, were counterbalanced between the participants through random allocation of the participants into one of the four task sets. The participants took the five sessions on separate weekdays. There was at least a twelve-hour interval between sessions, and the whole study was finished within 14 days. The duration of each session was about 40 min, and the total study duration was approximately 3 h and 20 min. Completion of EPELI in the first session was a prerequisite for partaking in the other sessions.

### 2.3. Word List Learning task

**Figure 2.**
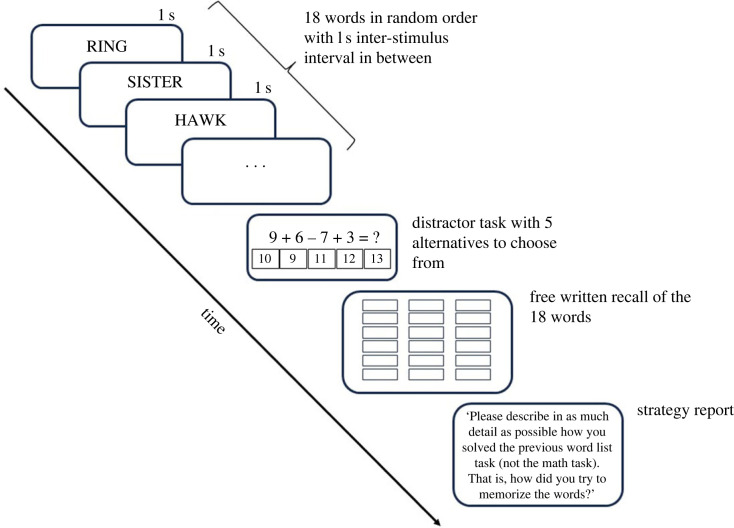
The structure of a task block in the Word List Learning task. Altogether three blocks were presented to the participants.

The present task of interest is an episodic memory and learning task adopted from Waris *et al*. [11]. Thus, it represents a commonly employed list learning format similar to, for example, the Rey Auditory Verbal Learning Task and enables the tracking of a participant's learning curve. It consists of an 18-word list of common nouns that Waris *et al*. [11] selected from the MRC Psycholinguistic Database with the following search criteria: (1) nouns according to the SOED database [25], (2) 4–6 letters long, (3) consisted of 1–3 syllables, (4) had a Kučera–Francis [26] written frequency above 0, and (5) had concreteness and imageability ratings of 558 or more (i.e. at least 1 s.d. above the mean). This resulted in the pool of 444 words that was next narrowed down to 283 high-frequency words as defined by Zipf frequency values of four or more [27] by using the SUBTLEX-US corpus [28]. Next, Waris and colleagues randomly selected two sets of 18 common nouns from this final word pool. In this study, we used one of the two lists (table 2). Our task was in all respects identical to the one used by Waris *et al*. [11] with one exception: instead of five presentation/free recall cycles of the 18 stimuli, the present shortened version included three cycles or task blocks. Shortening of the task avoided also any risk for ceiling effects: figure 1*a* in Waris *et al*. [11] shows that after three blocks, their participants' average performance was *ca* 13 words, well below the maximum of 18 words. The participants’ task was to try to memorize as many words as possible. The same 18 words were presented in each of the three blocks, but for each presentation, the order of the items was randomized. The structure of a task block is illustrated in figure 2.

**Table 2.** The stimuli of the current Word List Learning task taken from Waris *et al*. [11] (table 2, real words list 1).

word list	PALACE, ISLAND, STREET, HILL, POCKET, SISTER, TRASH, SOAP, TOOTH, POOL, TOWER, FLOWER, RING, NEEDLE, SWEAT, BOOT, HAWK, BLOOD

The words were shown on-screen one at a time for one second, separated by an inter-stimulus interval of one second during which the screen went blank. After the final word in a list had been shown, the participants were asked to solve a distractor task that appeared on-screen. The distractor tasks were multiple-choice arithmetical tasks (e.g. 9 + 6 − 7 + 3 = ?) intended to minimize the contribution of working memory to recall performance.

After the distractor task, the response screen was displayed, and the participants were instructed to type in the words they could recall one at a time in any order. Non-letter characters or spaces were permitted before or after the word, but otherwise the words had to be typed correctly. The dependent variable was the number of correctly recalled words per list.

After recalling words for a task block, the participants wrote their response to the following open-ended strategy query: ‘Please describe in as much detail as possible how you solved the previous word list task (not the math task). That is, how did you try to memorize the words?’.

### 2.4. Strategy coding for the Word List Learning task

Two independent raters coded each open-ended strategy report on three variables: the first reported strategy type (the primary strategy), the total number of specific strategy details given (be they for one or more strategy types), and the total number of strategy types reported. For each participant, there were 3 separate strategy reports for the Word List Learning task, i.e. one after each block. The first reported strategy was coded into one of 8 different strategy categories based on our earlier work on the classification of open-ended memory strategy reports [7,8,12], the same system that Waris *et al*. [11] employed. The categories used for coding Word List Learning task strategy responses were the following: No explicit strategy use, Rehearsal/Repetition, Grouping, Association, Visualization, Selective focus, Narrative^1^ and Other strategy. Electronic supplementary material, table S1, gives the detailed coding scheme with concrete examples.

Besides these primary strategy categories, we followed Waris *et al*. [11] by examining strategy use with a broader three-category classification of strategies taken from Fellman *et al*. [8]. Here, the strategies Rehearsal/Repetition and Selective focus were classified as a Maintenance strategy, whereas Grouping, Visualization, Association, Narrative and Other strategy were classified as a Manipulation strategy (the memoranda are manipulated in one way or another to facilitate recall). No strategy remained a single class of its own. After investigating the qualitative features of the strategy reports coded as Other strategy, we decided to lump Other strategy together with Manipulation instead of Maintenance, contrary to what Waris *et al*. [11] had done. This decision was made because the great majority of those reports included manipulation of the to-be-remembered material, such as using the words for forming sentences or linking the words together (e.g. ‘remembered a sentence in my head to remember as many words as possible’; ‘I tried to make them link together’).

Also, in line with Waris *et al*. [11] we coded the level of detail (LoD) in the strategy reports. A detail was defined as a specific strategy feature in the response and one point was given for each mentioned feature. In contrast to the Waris *et al*. [11] study, a detail point was not given for a reported specific strategy, but only for a reported specific feature of a strategy (see the coding scheme in the electronic supplementary material, table S1), and we left out no strategy users from the analysis. This was done to prevent a confound between the variables primary strategy use and the level of detail.

The third strategy measure that we coded, the total number of strategy types in a strategy report for each block, was a new variable that Waris *et al*. [11] had not used. The idea was that this variable could provide further information on participants' abilities to spontaneously generate and apply memory strategies.

Following the independent coding, we assessed the interrater reliability for the strategy coding in the Word List Learning task by unweighted kappa (*κ*) for the first reported strategy type and with linearly weighted kappa (*κ*_w_) for the total number of specific strategy details and the total number of different strategy types used. The data for these analyses comprised all participants including the ADHD group (total *n* = 328; the independent coders M.L. and T.E. were blinded to the group membership of the participants). The kappa coefficient was *κ* = 0.74 for the first reported strategy type, *κ*_w_ = 0.80 for the total number of specific strategy details and *κ*_w_ = 0.77 for the total number of different strategy types used. As all the reliability coefficients suggested substantial agreement, the raters continued with a subsequent consensus meeting where the discrepancies in their codings were discussed and solved.

### 2.5. Bayesian analytical approach

Regarding the three main findings by Waris *et al*. [11], our analytical approach was identical with one exception described below. As in their study, we employed Bayesian factors (BFs) using the ‘BayesFactor’ package [29] on R version 4.0.0 [30], but also JASP version 0.17.1. With this approach, the evidence either for the null hypothesis (H_01_) or for the alternative hypothesis (H_10_) is contested on a continuous scale. A BF of 1 indicates perfect ambiguity, whereas a BF above or below 1 provides evidence for the H_10_ or H_01_, respectively. Regarding the interpretation of the BFs, the guidelines put forth by Kass & Raftery [31] were followed: BFs between 1 and 3 represent ‘weak evidence’, BFs between 3 and 20 show ‘positive evidence’, BFs between 20 and 150 indicate ‘strong evidence’, and BFs greater than 150 are taken as ‘very strong evidence’. When relevant, we also report estimates of between-group mean differences using a posterior distribution with 10 000 iterations coupled with their 95% credible intervals formed from the highest density interval (HDI) distribution. In each BF analysis, we employed the default prior setting (i.e. Cauchy distribution using a scaling factor *r* = 0.707). As the Word List Learning task consists of consecutive blocks, LME [32] models were used whenever possible. In the present models, participants were treated as the crossed-random effect, and Block that was coded as a linear contrast represented always one of the fixed effects.

The one exception to the analyses conducted by Waris *et al*. [11] concerned their second finding, namely increased stability of strategic choices when the task advances. As their finding was purely descriptive, here we chose to test with a Bayesian binomial test whether the number of strategy changers decreased from the first to the second block transition. Moreover, as noted above, we coded and analysed a new strategy-related variable, the total number of strategy types in a strategy report for each block, to examine possible changes in that variable across the three blocks.

Outlier analysis was conducted in the same way as in Waris *et al*. [11]. We screened task performance for univariate outliers using the summed recall score across the three blocks as the dependent variable. Univariate outliers were defined as scores three times the interquartile range above or below the first or the third quartile. One such outlier was identified and excluded.

## 3. Results

### 3.1. General findings on learning progress and strategy use in the task

**Figure 3.**
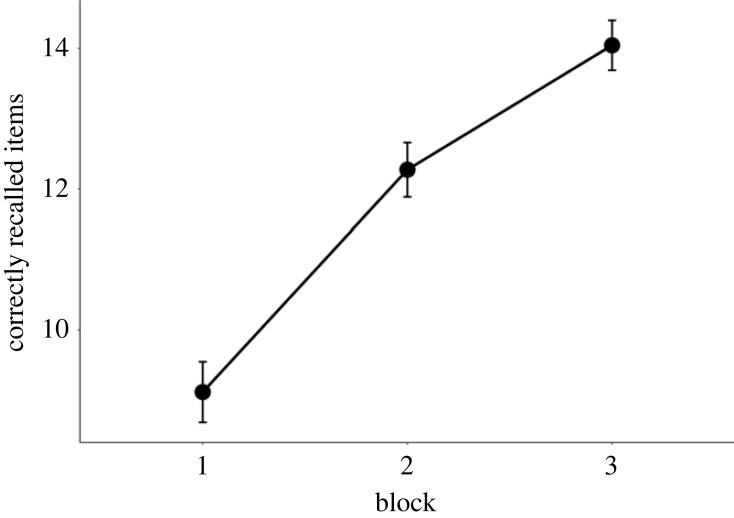
Average number of correctly recalled words across the three blocks. Whiskers in this and the following figures represent 95% confidence intervals.

As expected, the results showed very strong evidence for a main effect of block (*M*_diff_ = 2.46, 95% HDI = [2.31, 2.60], BF_10_ > 150 ± 0.69%), indicating that recall performance improved across the task blocks. As depicted in figure 3, the improvement pattern was more or less linear across the blocks.

Strategy use was prevalent in the task, and a clear majority of the participants reported using a strategy already in the first block (table 3).

**Table 3.** Percentage of participants using different strategy types across the three blocks.

strategy	block 1	block 2	block 3
no strategy	11.0	7.18	11.01
maintenance			
rehearsal/repetition	33.97	25.84	20.1
selective focus	0	20.1	25.84
manipulation			
grouping	10.53	9.09	9.1
association	8.61	5.26	3.83
visualization	18.66	14.83	13.88
narrative	9.57	10.05	9.09
other strategy	7.66	7.66	7.18

### 3.2. The first hypothesis: strategy use is associated with objective memory performance

#### 3.2.1. Primary strategy type and test performance

The first main finding by Waris *et al*. [11] that we attempted to replicate was that strategy use in word list learning is linked to better objective task performance. For this purpose, we grouped the participants based on their open-ended strategy reports. Similarly to Waris *et al*. [11], we used the broader categories described above (Maintenance, Manipulation/Other strategy, No strategy). The participants were grouped according to what primary strategy category they had reported most frequently during the three blocks. If a participant reported using different strategies an equal number of times, the most sophisticated strategy was chosen (No strategy < Maintenance < Manipulation/Other [8]). This resulted in the following group sizes: No strategy, *n* = 16; Maintenance, *n* = 89; Manipulation/Other, *n* = 104.

**Figure 4.**
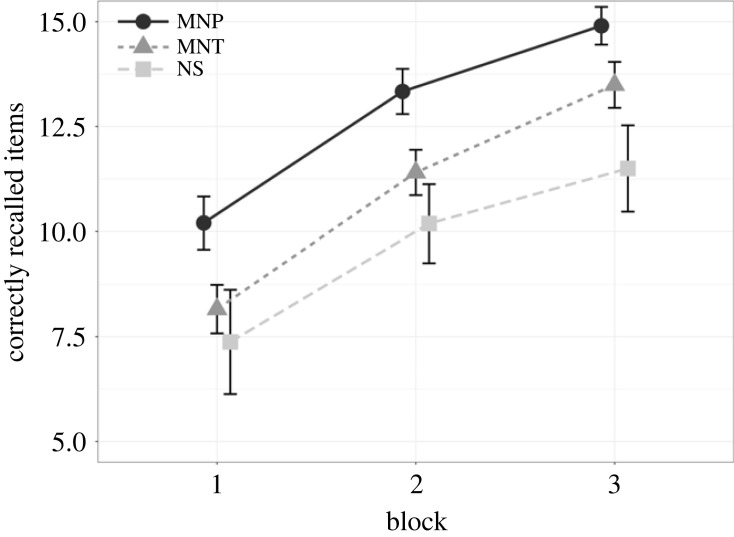
Average number of correctly recalled words in users of Manipulation/Other strategy (MNP), Maintenance strategy (MNT), and No strategy (NS) across the three blocks.

LME models were computed to test whether strategy type was associated with recall performance across blocks. We performed pairwise comparisons between each strategy type (table 4 and figure 4). There was very strong evidence for a main effect of strategy type between Manipulation/Other strategy and Maintenance, indicating better performance in users of the former category across the task. This was true also for the comparison between Manipulation/Other strategy and No strategy. In turn, Maintenance versus No strategy comparison did not reveal evidence for a difference. None of the comparisons showed evidence for strategy type × block interaction.

**Table 4.** Pairwise comparisons of Word List Learning performance between users of Manipulation/Other strategy, Maintenance strategy, and No strategy. MNP/OTHER: Manipulation or Other strategy; MNT: Maintenance; NS: No strategy. HDI: highest density interval of the posterior distribution; BF: Bayesian factor. Estimates are the mean group differences from 10 000 samples of the posterior distribution. Bolded values are the results that provide evidence for the alternative hypothesis.

effect	MNP/OTHER versus MNT^a^		MNP/OTHER versus NS^a^		MNT versus NS^b^	
	*M*_Diff_ [95% HDI]	BF ± error (%)	*M*_Diff_ [95% HDI]	BF ± error (%)	*M*_Diff_ [95% HDI]	BF ± error (%)
strategy	1.72 [1.01–2.4]	**BF_10_ > 150** ± **3.54**	2.91 [1.61–4.2]	**BF_10_ > 150** ± **19.1**8	1.06 [−0.11–2.28]	BF_10_ = 1.81 ± 3.35
block	2.5 [2.34–2.66]	**BF_10_ > 150** ± **1.79**	2.19 [1.9–2.5]	**BF_10_ > 150** ± **19.2**	2.36 [2.11–2.59]	**BF_10_ > 150** ± **3.17**
interaction	−0.31 [−0.62–−0.01]	BF_01_ = 3.33 ± 2.41	0.29 [−0.33–0.9]	BF_01_ = 14.29 ± 19.25	0.6 [0.08–1.07]	BF_01_ = 1.18 ± 3.5

^a^Positive values represent greater performance in the MNP/OTHER.

^b^Positive values represent greater performance in the MNT.

In sum, the analyses described above replicated the finding by Waris *et al*. [11] that the use of a Manipulation strategy is associated with superior Word List Learning performance. However, their finding on a performance difference between Maintenance strategy and No strategy users was not replicated.

#### 3.2.2. Level of strategy detail and test performance (table 5)

LME model for the effect of LoD across the blocks revealed strong evidence for a main effect of LoD (*M*_diff_ = 0.18, 95% HDI = [0.10, 0.27], BF_10_ = 80.28 ± 0.77%) so that higher LoD scores were associated with a better memory performance. We observed no evidence for a block × LoD interaction (*M*_diff_ = −0.07, 95% HDI = [−0.13, −0.02], BF_01_ = 1.04 ± 0.65%). These findings replicate fully the corresponding results obtained by Waris *et al*. [11].

**Table 5.** Average number of correctly recalled words across the three blocks among strategy users by level of detail and number of different strategy types used. LoD, level of detail; STRAT, number of different strategy types used.

	block 1	block 2	block 3
	*M* (s.d.)	*M* (s.d.)	*M* (s.d.)
LoD = 0	8.78 (2.86)	11.94 (2.67)	13.87 (2.53)
LoD ≥1	10.62 (3.37)	13.84 (2.88)	15.20 (2.13)
STRAT = 1	9.22 (3.35)	12.06 (2.79)	13.85 (2.61)
STRAT > 1	9.68 (2.69)	13.06 (2.85)	14.82 (2.2)

#### 3.2.3. Total number of strategy types and test performance (table 5)

We observed only weak evidence for a main effect of the number of strategy types on memory performance (*M*_diff_ = 0.39, 95% HDI = [0.09, 0.68], BF_10_ = 1.52 ± 1.23%), indicating that this variable was not associated with Word List Learning scores. There was also positive evidence against an interaction between the number of strategy types and block (*M*_diff_ = −0.10, 95% HDI = [−0.37, 0.17], BF_01_ = 14.29 ± 1.03%). As figure 6*b* indicates, most strategy users reported only a single strategy.

### 3.3. The second hypothesis: strategy changes become less frequent across the task blocks

**Figure 5.**
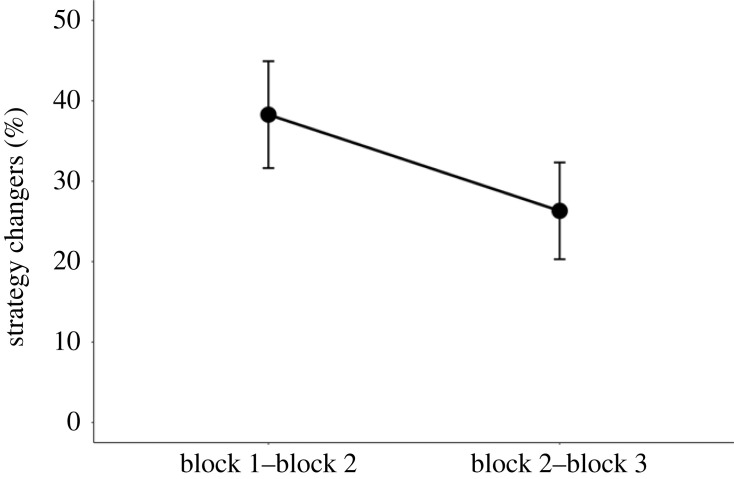
Percentage of strategy changers from one block to another.

This analysis concerned the number of participants who changed their primary strategy type when moving from one block to another. The hypothesis based on Waris *et al*. [11] was that this number is higher in the first block transition (block 1 → block 2) than in the second one (block 2 → block 3), as strategy use should begin to stabilize quickly after the initial strategy generation stage. We cross-tabulated the participants and found that the number of participants who changed their strategy in the first but not in the second block transition was 54. This contrasted with 29 participants who exhibited the opposite pattern. A hundred participants did not change their strategy in either the first or the second block transition, and 26 changed strategy in both transitions. A Bayesian binomial test with JASP (version 0.17.2.1) provided positive evidence for the hypothesis that the number of strategy changers decreased from the first to the second block transition (BF_10_ = 5.96). Thus, the finding of a decreased rate of strategy changers across the task blocks was replicated, albeit the present shortened version had three task blocks and thus only two block transitions instead of five blocks and four transitions. The percentages of all strategy changers in the two block transitions are shown in figure 5.

### 3.4. The third hypothesis: strategy use increases during the first two task blocks

**Figure 6.**
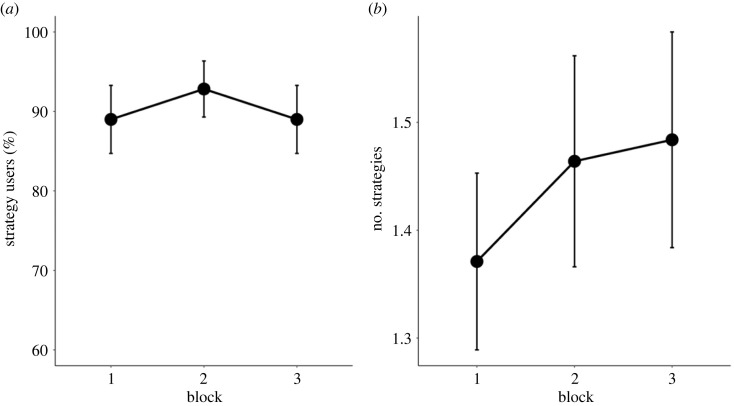
(*a*) Percentage of strategy users across blocks. (*b*) Average number of different strategy types used per block among strategy users in that block.

Following the *post hoc* finding by Waris *et al*. [11], we hypothesized that strategy use increases during the first two task blocks. No evidence for a main effect of block was observed (*M*_diff_ = 0.04, 95% HDI = [−0.01, 0.07], BF_01_ = 2.00 ± 1.21%) (figure 6*a*). Thus, this finding by Waris *et al*. [11] was not replicated. However, one should note that their finding was based on an analysis that included the first two blocks of both the real word and the pseudoword task. Moreover, their fig. 1*b* indicates that the increase in strategy use was less marked in the real word task, even though there was no evidence for a task × block interaction. Thus, to obtain a direct comparison, we re-analysed the block 1–block 2 data of Waris *et al*. [11] by including only the real word task. This analysis gave only weak support for an increase in strategy use (BF_10_ = 1.45). Thus, the present hypothesis was misguided as far as only the real word learning task is concerned. Figure 6*a* suggests a slight average increase in strategy use from block 1 to block 2, followed by a similar decrease at block 3. The pattern for these three blocks is as such very similar as in fig. 1*b* in Waris *et al*. [11].

Finally, we also examined whether the number of primary strategies showed any change across the three task blocks (figure 6*b*). The results revealed weak evidence for the null hypothesis (*M*_diff_ = 0.06, 95% HDI = [0.02, 0.11], BF_01_ = 1.58 ± 1.69%), indicating that the number of strategies remained unchanged over the three task blocks. When inspecting the same relationship by excluding the third block, we again observed weak evidence for the null hypothesis (*M*_diff_ = 0.10, 95% HDI = [0.01, 0.20], BF_10_ = 1.04 ± 1.24%), indicating no evidence for an increase in the number of strategies from the first to the second block. As noted earlier, most strategy users reported only one strategy, so the variability on this measure was limited.

## 4. Discussion

Given the scarcity of detailed studies on spontaneous strategy use during episodic memory task performance, this study set out to replicate the main findings of Waris *et al*. [11] who examined the strategy–performance relationships and the evolvement of strategies in a Word List Learning task. Their three main findings were: strategy use is associated with better objective task performance, strategy choices become more stable during the first block transitions, and strategy use increases during the first two task blocks. By using a shortened form with one of the stimulus sets employed in the original study, we managed to replicate the first finding partly and the second finding fully. The third finding turned out to be misguided as the original analysis was based on a summative effect of both word and pseudoword learning, not on the real word condition that we employed. In what follows, we discuss these findings and their implications in more detail.

The finding that spontaneous strategy use is associated with enhanced episodic memory performance has been observed also in earlier studies [15,33,34] and highlights the importance of strategies in understanding factors that underlie the considerable inter-individual differences in memory performance. More specifically, the present results replicated the finding by Waris *et al*. [11] that the family of strategies involving manipulation of memoranda in mind was related to superior word recall as compared to Maintenance strategies (Rehearsal/Repetition and Selective Focus) or to No strategy. The Manipulation strategies, here including Grouping, Visualization, Association, Narrative and Other Strategy, arguably represent the cognitively most advanced mnemonics in the present repertoire, requiring executive control. Their spontaneous use in Word List Learning is also quite common. In Waris *et al*. [11], they were used in the first three blocks by 46% (block 1), 37% (block 2), and 35% (block 3) of the participants (to make their percentages directly comparable to ours, Other strategy users are included in these figures). In the present replication study, the corresponding percentages were even higher (55% for block 1, 47% for block 2, and 43% for block 3), pointing to a certain variability in frequencies of strategic choices from one adult sample to another. One finding by Waris *et al*. [11] that did not replicate was that in their study, the use of Maintenance strategies was linked to better word recall than No strategy. Thus, the benefit from using these cognitively more simple strategies is less certain. In a review concerning working memory task performance, Oberauer [35] also questioned the facilitatory role of simple rehearsal.

The other two findings of Waris *et al*. [11] that we attempted to replicate concerned dynamic changes in strategy use during the rather short time span it took to perform the Word List Learning task. The first one of these findings that we managed to replicate was that strategic choices became more stable when the task advanced. In Waris *et al*. [11], this was only a descriptive finding, but here we could ascertain it statistically by comparing the rates of strategy changers in the first versus the second block transition. The decrease in the rates of strategy changers during block transitions speaks for a dynamic process where task-initial strategy generation and adjustment is followed by a more stable use of a chosen strategy. Regarding Word List Learning, spontaneous strategy shifts from more superficial initial strategies to deeper and more effective ones have been reported in some earlier studies [16–18]. A possible counterargument to the present interpretation is that task-initial strategy changes could be a result of the task structure where the same items are presented repeatedly. However, the clustering of strategy shifts especially in the task-initial phases seems to be a more general phenomenon, as we have found it also in a prospective/episodic memory task with constantly changing task items [36], and in a continuous working memory updating task [12]. Theoretically, initial strategy shifts followed by a gradual strategy stabilization in all these different memory tasks fits well to the cognitive skill learning view [19]. According to this view, demanding non-routine tasks activate the metacognitive and executive control systems needed for strategy generation and implementation/monitoring, after which task routine starts to gradually develop [19]. Being a general framework, the cognitive skill learning view does not take a stance on the time span of these hypothetical stages in specific tasks, but the results from three different memory paradigms, Word List Learning task [11], a complex virtual reality prospective/episodic memory task [36], and a continuous n-back working memory updating task [12], suggest that the most intensive strategy generation and adaptation stage is rather short-lived, being most prominent in the first task blocks. These memory tasks are, after all, quite straightforward (albeit cognitively demanding), and it may very well be that strategy generation and adaptation would take longer for example in complex problem-solving tasks.

The third finding by Waris *et al*. [11], increase in strategy use during the first two task blocks, failed to replicate. However, as we describe in the Results section, this hypothesis turned out to be misguided, as a *post hoc* analysis of the original data showed that the effect was not present in the real word condition that was the focus of this replication attempt. Thus, one might rather turn the argument the other way round and note that the lack of positive evidence for such an increase was replicated. A plausible reason for a lack of increase in strategy use is the fact that the percentage of strategy users was high already in the first real word task block, leaving limited room for a further increase. The percentages of strategy users in the first three blocks were 85%–91%–85% in Waris *et al*. [11], and 89%–92%–89% in the present replication study. As such, these values are quite close to each other. In the block-by-block strategy analysis of the working memory updating task where an initial increase in strategy use was reported [12], there was much more room for improvement, as the percentage of strategy users in the first task block was about 50% and going up to *ca* 65% in the second block in both n-back experiments. Regarding the new strategy variable that we included in this study, the number of strategy types employed, the rates were at a low (mostly single strategy) and stable level throughout the blocks. All in all, possible increase in strategy use at the task-initial stages remains an elusive phenomenon that may require specific task conditions to appear. As compared to strategy change discussed above, the strategy increase variable is also much more limited in scope. Strategy change encompasses three types of changes, shifts between different primary strategy types, changes from a primary strategy to no strategy, and changes from no strategy to a primary strategy, while strategy increase concerns only the last type of change.

An important theoretical aim of the study by Waris *et al*. [11] was to test the cognitive routine framework proposed by Gathercole *et al*. [20]. This framework is closely linked to cognitive skill learning discussed above and was developed to account for the very limited transfer observed in working memory training studies (e.g. [37,38]). However, it has implications for memory task practice in general. According to this framework, repeated practice with a memory task leads to development of new cognitive routines (i.e. strategies) under the condition that the trained task is unfamiliar. In turn, a familiar memory task like the present one where participants learn a list of high-frequency real words should not trigger strategy development. Gathercole *et al*. [20] note that for a familiar task like verbal serial recall, novel material-specific strategies would be adopted only ‘under conditions of extensive and prolonged practice’ (p. 23), which is very different from the present single-session setup. Waris *et al*. [11] contrasted the cognitive routine framework to a competing hypothesis that they coined as the task demand hypothesis. This alternative hypothesis states that besides novelty, also task demands play a role in the spontaneous adoption of strategies. Thus, strategies can be generated also when faced with a familiar task if the task is demanding enough. This is partly in line with Belmont & Mitchell [39] who proposed that cognitive tasks which participants perceive as moderately difficult (not easy or very difficult) are more likely to elicit strategic behaviour. Considering these two hypotheses, the present findings are in line with the task demand hypothesis, as the rather demanding 18-item real word learning task triggered frequent strategy use right from the start and exhibited strategy adjustments (changes of strategy) especially during the first two task blocks. However, as noted by Waris *et al*. [11], the task demand hypothesis is complementary rather than opposite to the cognitive routine framework. Thus, one can conclude that both novelty and task demands affect strategy use when performing a cognitive task.

The same general limitations as those of Waris *et al*. [11] concern also the present study. Firstly, both are online studies conducted with anonymous participants, with no control over the conditions under which the participants took the task. However, the instructions emphasized that the participants should ensure that they work alone in a quiet space. Previous studies have also shown a close correspondence between the cognitive task effects obtained in online and laboratory experiments (e.g. [40,41]). Secondly, strategy information was based on introspective reports that may not cover all relevant strategic behaviours that the participants employed. Nevertheless, the fact that self-reported strategies were strongly associated with actual recall performance speaks for their relevance in strategy research. In future studies, one could look for opportunities to gather simultaneously both subjective and objective strategy data (e.g. degree of semantic clustering of recalled words in free recall). Thirdly, it is worth noting that the present data on the relationships between strategy use and memory performance are correlative. However, performance improvements in previous studies where participants received memory strategy instructions speak for a causal relationship between strategy employment and performance (e.g. [3,4,7–10,42–44]).

## 5. Conclusion

In summary, the present study replicated partly the finding by Waris *et al*. [11] that self-reported spontaneous strategy use is associated with superior recall performance when learning real words. This was shown here for the Manipulation strategies but not for the simpler Maintenance strategies. Moreover, we replicated fully the finding that changes in strategy use clustered especially to the first two task blocks. These findings were taken as support to the view that when faced with a demanding memory task, even a familiar one, adult participants are prone to use and adjust mnemonic strategies right from the start. Thus, complex memory tasks should not be considered as straightforward capacity measures, because the use or non-use of an effective strategy can make a considerable difference in task outcomes. This harks back to the notion by Atkinson & Shiffrin [45] in their seminal 1968 paper: any theory of human memory that aims at generality must include control processes such as rehearsal, coding and search strategies. The more general interpretation of the present findings is that performance on a demanding cognitive task represents cognitive skill learning where task-specific strategies are adopted. In memory tasks that are quite straightforward, the first stages of cognitive skill learning, strategy generation and adaptation, appear to be short-lived. These hidden task-initial dynamics of cognitive task performance become visible only when detailed block-by-block analyses of strategy use are employed.

## Data Availability

Data and R-code to reproduce the analyses are available through the Open Science Framework at https://osf.io/pdxsr/ [46]. Supplementary material is available online [47].

## Footnotes

1 This category was created afterwards, as a second look at the unclassified strategy responses indicated several instances of an explicit mention of creating a story in order to remember the words.
